# Combination of cyclosporine and erythropoietin improves brain infarct size and neurological function in rats after ischemic stroke

**DOI:** 10.1186/1479-5876-9-141

**Published:** 2011-08-24

**Authors:** Chun-Man Yuen, Cheuk-Kwan Sun, Yu-Chun Lin, Li-Teh Chang, Ying-Hsien Kao, Chia-Hung Yen, Yung-Lung Chen, Tzu-Hsien Tsai, Sarah Chua, Pei-Lin Shao, Steve Leu, Hon-Kan Yip

**Affiliations:** 1 Division of Trauma, Department of Surgery, Kaohsiung Chang Gung Memorial Hospital and Chang Gung University College of Medicine, Kaohsiung, Taiwan; 2 Department of Emergency Medicine, E-Da Hospital, I-Shou University, Kaohsiung, Taiwan; 3 Department of Medical Research, E-Da Hospital, I-Shou University, Kaohsiung, Taiwan; 4 Basic Science, Nursing Department, Meiho University, Pingtung, Taiwan; 5 Department of Life Science, National Pingtung University of Science and Technology, Pingtung, Taiwan; 6 Division of cardiology, Department of Internal Medicine, Kaohsiung Chang Gung Memorial Hospital and Chang Gung University College of Medicine, Kaohsiung, Taiwan; 7 Graduate Institute of Medicine, College of Medicine, Kaohsiung Medical University, Kaohsiung, Taiwan; 8 Center for Translational Research in Biomedical Sciences, Kaohsiung Chang Gung Memorial Hospital and Chang Gung University College of Medicine, Kaohsiung, Taiwan

## Abstract

**Background:**

This study tested the superiority of combined cyclosporine A (CsA)-erythropoietin (EPO) therapy compared with either one in limiting brain infarction area (BIA) and preserving neurological function in rat after ischemic stroke (IS).

**Methods:**

Fifty adult-male SD rats were equally divided into sham control (group 1), IS plus intra-peritoneal physiological saline (at 0.5/24/48 h after IS) (group 2), IS plus CsA (20.0 mg/kg at 0.5/24h, intra-peritoneal) (group 3), IS plus EPO (5,000IU/kg at 0.5/24/48h, subcutaneous) (group 4), combined CsA and EPO (same route and dosage as groups 3 and 4) treatment (group 5) after occlusion of distal left internal carotid artery.

**Results:**

BIA on day 21 after acute IS was higher in group 2 than in other groups and lowest in group 5 (all p < 0.01). The sensorimotor functional test showed higher frequency of left turning in group 2 than in other groups and lowest in group 5 (all p < 0.05). mRNA and protein expressions of apoptotic markers and number of apoptotic nuclei on TUNEL were higher in group 2 than in other groups and lowest in group 1 and 5, whereas the anti-apoptotic markers exhibited an opposite trend (all p < 0.05). The expressions of inflammatory and oxidized protein were higher in group 2 than in other groups and lowest in group 1 and 5, whereas anti-inflammatory markers showed reversed changes in group 1 and other groups (all p < 0.05). The number of aquaporin-4+ and glial fibrillary acid protein+ stained cells were higher in group 2 as compared to other groups and lowest in groups 1 and 5 (all p < 0.01).

**Conclusion:**

combined treatment with CsA and EPO was superior to either one alone in protecting rat brain from ischemic damage after IS.

## Background

Despite current advances in medicine and implementation of the state-of-the-art management guidelines, ischemic stroke (IS) remains the leading cause of death in the industrial countries regardless of etiologies [1-4]. Indeed, this unsavory clinical problem has vexed neurologists for decades. Not only the death but also the high incidence of severe neurological impairment after IS with permanent disability [5] that cause a tremendous social economic burden worldwide. Although growing data indicate that the newly developed thrombolytic therapy offers a promising treatment option for some patients with acute IS early after the onset of symptoms [6,7], its clinical application is impeded by major limitations [7-10]. Besides, thrombolytic therapy has been reported to be associated with a relatively high incidence of intracranial hemorrhage [10,11] contributing to its notable mortality and morbidity. Accordingly, the treatment of acute IS patients remains problematic. Therefore, finding a safe and effective therapeutic regimen for patients following acute IS, especially for those unsuitable for thrombolytic therapy, is of utmost importance for physicians.

Not only has erythropoietin (EPO) therapy been reported to enhance erythropoiesis in the treatment of anemia, but it has also been shown to alleviate ischemia-related organ dysfunction through anti-ischemic and cellular protective effects [12-15]. Our recent studies [16,17] have further shown that EPO therapy remarkably improves neurological impairment in rat IS model and clinical outcome in patients after acute IS. Additionally, accumulating evidence from animal models indicates that not only does cyclosporine A (CsA) possess immunosuppressive properties, but it is also a potent inhibitor of mitochondrial permeability transition pore (mPTP) that plays an important role in attenuating ischemia-reperfusion injury [18-20]. Recently, a clinical observational study [21] and an experimental investigation using a mini-pig animal model [22] demonstrated that administration of CsA after acute ST-segment elevation myocardial infarction (STEMI) effectively limited left ventricular infarct size. However, whether combined therapy with CsA and EPO will maximize the anti-ischemic effect and further improve outcome after acute IS remains uncertain. In view of the fact that there is no effective therapy for the majority of patients with acute IS and that both EPO and CsA have been shown to offer therapeutic benefit to this patient population, this study investigated whether combined therapy with these two drugs was superior to either one alone in reducing brain infarction and improving neurological function in a rat acute IS model.

## Methods

### Ethics

All animal experimental procedures were approved by the Institute of Animal Care and Use Committee at our institute and performed in accordance with the Guide for the Care and Use of Laboratory Animals (NIH publication No. 85-23, National Academy Press, Washington, DC, USA, revised 1996).

### Animal Model of Acute Ischemic Stoke and Corner Test

The protocol and procedure of using a rodent model of acute IS has been described in details in our recent report [23]. Adult male Sprague-Dawley rats, weighing 300-325 g (Charles River Technology, BioLASCO Taiwan Co., Ltd., Taiwan) were utilized in the current study. All animals were anesthetized by chloral hydrate (35 mg/kg i.p.) and placed in a supine position on a warming pad at 37°C. After exposure of the left common carotid artery (LCCA) through a transverse neck incision, a small incision was made on the LCCA through which a nylon filament (0.28 mm in diameter) was carefully advanced into the distal left internal carotid artery for occlusion of left middle cerebral artery (LMCA) to induce brain infarction of its blood-supplying area. The nylon filament was removed three hours after occlusion, followed by closure of the muscle and skin in layers. The rats were then placed in a portable animal intensive care unit (ThermoCare^®^) for 24 hours. The sensorimotor functional test (Corner test) was done for each rat at baseline and on day 1 (24 h after procedure), 3, 7, 14, and 21 after acute IS induction as we recently described [16,23]. Briefly, the rat was allowed to walk through a tunnel and then into a corner, the angle of which was 60 degrees. To exit the corner, the rat could turn either to left or right. The results were recorded by a technician who was blind to the study design. This test was repeated 10 to 15 times with at least 30 seconds between each trial. We recorded the number of right and left turns from 10 successful trials for each animal and used the results for statistical analysis.

### Treatment Protocol

Ten sham-operated healthy rats served as normal controls (group 1). The other 40 rats with acute IS were equally divided into IS plus intra-peritoneal 1.0 mL physiological saline (at 0.5, 24 and 48 hour after IS) (group 2, n = 10), IS plus CsA (20.0 mg/kg at 0.5 and 24 hour, intra-peritoneal) (group 3, n = 10), IS plus EPO (5,000 IU/kg at 0.5, 24, and 48 hour, subcutaneous) (group 4, n = 10), and combined CsA (20.0 mg/kg at 0.5 and 24 hour, intra-peritoneal) and EPO (5,000 IU/kg at 0.5, 24 and 48 hour, subcutaneous) treatment (group 5, n = 10).

Two rats died in group 2 and one rat died in each other group (i.e. groups 3 to 5) during the procedure. For the purpose of this study, additional rats were added so that 10 animals in each group went through the whole study.

The dosage of EPO and the timing of treatment were based on previous literature and our recent report [16,24], whereas the dosage of cyclosporine and the treatment protocol were according to a previous report [25].

### Specimen Collection and Preparation for Individual Study

Rats in all groups were euthanized on day 21 after IS induction, and the brain of each rat was promptly removed and immersed in cold saline. For immunohistofluorescent (IHF) study, the brain tissue was rinsed with PBS, embedded in OCT compound (Tissue-Tek, Sakura, Netherlands) and snap-frozen in liquid nitrogen before being stored at -80°C. For immunohistochemical (IHC) staining, the brain tissue was fixed in 4% formaldehyde and embedded in paraffin. Additionally, the brain tissue of infarct area was collected for Western blot, real-time PCR, and oxidative stress analyses.

### Measurement of Brain Infarct Area

To evaluate the impact of CsA, EPO, and combined EPO and CsA treatment on brain infarction, coronal sections of the brain were obtained from six extra animals in groups 2 to 5 (n = 6 for each group) as 2 mm slices. Each cross section of brain tissue was then stained with 2% 3,5-Triphenyl-2H-Tetrazolium Chloride (TTC) (Alfa Aesar) for BIA analysis. The methodology has been described in details in our recent studies [16,23]. Briefly, all brain sections were placed on a tray with a scaled vertical bar to which a digital camera was attached. The sections were photographed from directly above at a fixed height. The images obtained were then analyzed using Image Tool 3 (IT3) image analysis software (University of Texas, Health Science Center, San Antonio, UTHSCSA; Image Tool for Windows, Version 3.0, USA). BIA was identified as either whitish or pale yellowish regions. Infarct region was further confirmed by microscopic examination. The percentages of infarct area were then calculated by dividing the area with total cross-sectional area of the brain.

All measurements (i.e. Corner test and assessment of BIA) were performed by a skillful senior technician blinded to the treatment and non-treatment groups.

### TUNEL Assay for Apoptotic Nuclei

For each rat, six sections of BIA were analyzed by an in situ Cell Death Detection Kit, AP (Roche) according to the manufacturer's guidelines. Three randomly chosen high-power fields (HPFs) (×400) were observed for terminal deoxynucleotidyl transferase-mediated 2'-deoxyuridine 5'-triphosphate nick-end labeling (TUNEL)-positive cells for each section. The mean number of apoptotic nuclei per HPF for each animal was obtained by dividing the total number of cells with 18.

### Immunofluorescent Staining

Frozen sections (4 μm thick) were obtained from BIA of each animal. The sections were fixed with 4% paraformaldehyde and permeated with 0.5% Triton X-100 and then incubated with antibodies against NeuN (1:1000, Millipore), GFAP (1:500, DAKO), PGC-1α (1:100, Santa cruz), and AQP4 (1:200, abcam) at 4°C overnight. Alexa Fluor488, Alexa Fluor568, or Alexa Fluor594-conjugated goat anti-mouse or rabbit IgG were used to localize signals. Sections were then counterstained with DAPI and observed with a fluorescent microscope equipped with epifluorescence (Olympus IX-40).

### Western Blot Analysis for Bax, Cytochrome C, Caspase 3, NADPH oxidase 1 (NOX-1), NOX-2, Inducible Nitric Oxide Synthase (iNOS), and Endothelial (e)NOS

Equal amounts (50 mg) of protein extracts were loaded and separated by SDS-PAGE using 12% acrylamide gradients. After electrophoresis, the separated proteins were transferred electrophoretically to a polyvinylidene difluoride (PVDF) membrane (Amersham Biosciences). Nonspecific sites were blocked by incubation of the membrane in blocking buffer [5% nonfat dry milk in T-TBS (TBS containing 0.05% Tween 20)] for overnight. The membranes were incubated with the indicated primary antibodies (Bax, 1:1000, abcam; Cytochrome C, 1:2000, BD; Caspase, 1:3000, abcam; NOX-1, 1:1500, Sigma; NOX-2, 1:500, Sigma; iNOS, 1:200, abcam; eNOS, 1:1000, 1:500, abcam; Actin, 1:10000, Chemicon) for 1 hr at room temperature. Horseradish peroxidase -conjugated anti-rabbit or anti-mouse immunoglobulin IgG (1:2000, Cell Signaling) was used as a second antibody for 1 hr at room temperature. The washing procedure was repeated eight times within 1h, and immunoreactive bands were visualized by enhanced chemiluminescence (ECL; Amersham Biosciences) and exposure to Biomax L film (Kodak). For purposes of quantitation, ECL signals were digitized using Labwork software (UVP).

### Protocol for RNA Extraction

Lysis/binding buffer (High Pure RNA Tissue Kit, Roche, Germany) 400 μL and an appropriate amount of frozen brain tissues were added to a nuclease-free 1.5 mL microcentrifuge tube, followed by disruption and homogenization of the tissue by using a rotor-stator homogenizer (Roche).

For each isolation, 90 mL DNase incubation buffer was pipetted into a sterile 1.5 mL reaction tube, 10 mL DNase I working solution was then added, mixed and incubated for 15 min at 25°C. Wash buffer I 500 mL was then added to the upper reservoir of the filter tube, which was then centrifuged for 15 seconds at 8,000*g*. Wash buffer II 300 mL was added to the upper reservoir of the filter tube, which was centrifuged for 2 min full-speed at approximately 13,000*g*. Elution Buffer 100 mL was then added to the upper reservoir of the filter tube. Finally, the tube assembly was centrifuged for 1 min at 8,000*g*, resulting in eluted RNA in the microcentrifuge tube.

### Real-Time Quantitative PCR Analysis

Real-time polymerase chain reaction was conducted using LighCycler TaqMan Master (Roche, Germany) in a single capillary tube according to the manufacturer's guidelines for individual component concentrations. Forward and reverse primers were each designed based on individual exons of the target gene sequence to avoid amplifying genomic DNA.

During PCR, the probe was hybridized to its complementary single-strand DNA sequence within the PCR target. As amplification occurred, the probe was degraded due to the exonuclease activity of Taq DNA polymerase, thereby separating the quencher from reporter dye during extension. During the entire amplification cycle, light emission increased exponentially. A positive result was determined by identifying the threshold cycle value at which reporter dye emission appeared above background. For normalization, the housekeeping gene Peptidyl-prolyl cis-trans isomerasa (Ppia, Cyclophilin A) was used as the reference gene.

### Oxidative Stress Reaction of BIA

The Oxyblot Oxidized Protein Detection Kit was purchased from Chemicon (S7150). The oxyblot procedure was performed according to the previous study [26]. The 2,4-dinitrophenylhydrazine (DNPH) derivatization was carried out on 6 μg of protein for 15 min according to manufacturer's instructions. One-dimensional electrophoresis was carried out on 12% SDS/polyacrylamide gel after DNPH derivatization. Proteins were transferred to nitrocellulose membranes which were then incubated in the primary antibody solution (anti-DNP 1:150) for 2 h, followed by incubation with second antibody solution (1:300) for 1 h at room temperature. The washing procedure was repeated eight times within 40 min. Immunoreactive bands were visualized by enhanced chemiluminescence (ECL; Amersham Biosciences) which was then exposed to Biomax L film (Kodak). For quantification, ECL signals were digitized using Labwork software (UVP). On each gel, a standard control sample was loaded.

### Statistical Analysis

Data were expressed as mean values (mean ± SD). Statistical analysis was adequately performed by analysis of variance, followed by Scheffe multiple-comparison post hoc test. SAS statistical software for Windows version 8.2 was utilized. (SAS institute, Cary, NC). A probability value < 0.05 was considered statistically significant.

## Results

### Effect of Combined CsA and EPO on Infarction Area and Neurological Function after Acute IS

**Figure 1:**
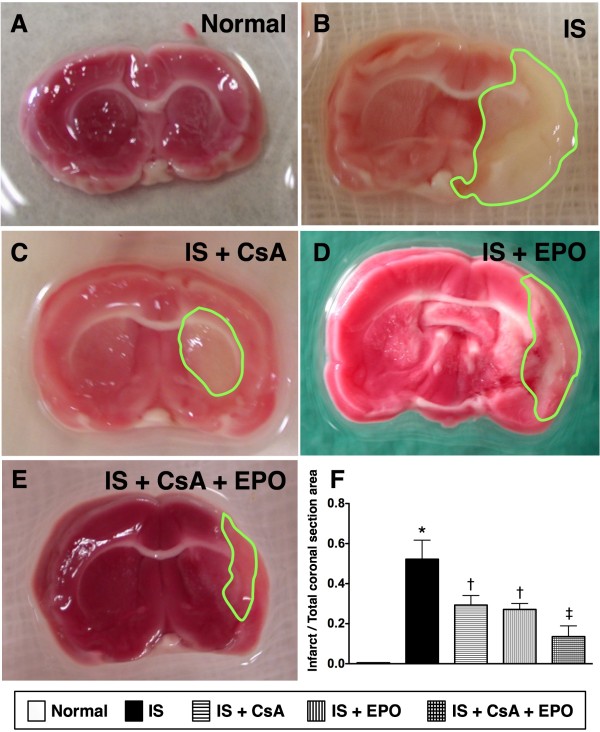
**Ratios of infarct area to total coronal sectional area using TTC staining**. **(A to E) **Identification of gross infarct area (green circles) in animals with **B) **ischemic stroke (IS) (group 2), **C) **IS + cyclosporine (CsA) (group 3), **D) **IS + erythropoietin (EPO) (group 4) and **E) **IS + combined CsA & EPO (group 5), respectively. **(F) **Significantly lower ratio of infarct area to total coronal sectional area in group 5 than in group 2, 3, and 4, and notably lower in group 3 and 4 than in group 2 (n = 6 for each group). * vs. other groups, p < 0.0001 (using ANOVA). Symbols (*, †, ‡) indicate significance (at 0.05 level) (by Scheffe multiple-comparison post hoc test).

The mortality rate [2 in group 2, 1 in each other group (i.e. groups 3 to 5)] did not statistically differ among groups 2 to 5 (p = 0.413). TTC staining of brain tissues on day 21 after acute IS showed notably reduced BIA in IS animals treated with CsA (group 3) and EPO (group 4) than in IS animals without treatment (group 2), and further reduced after combined therapy with CsA and EPO (group 5) than in group 3 and group 4 (Figure 1).

**Figure 2:**
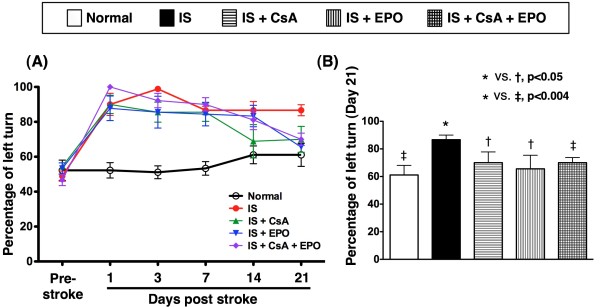
**Assessment of neurological function with Corner test**. **A) **The results of Corner test (n = 10) on day 0, 1, 3, 7, 14, and 21 after acute IS, showing a steady state of neurological functional impairment on day 3 to 14 following acute IS in group 2, 3, 4, and 5. **B) **Significant improvement in neurological function noted in group 3, 4, and 5 compared with group 2 on day 21 after acute IS, and further improvement observed in group 5 compared with group 3 and 4. * vs. other groups, p < 0.001 (at day 21). Symbols (*, †, ‡) indicate significance (at 0.05 level) (by Scheffe multiple-comparison post hoc test).

Corner test showed that, as compared with group 2, a transient improvement in neurological function was noted in groups 3 to 5 on day 3 after acute IS (Figure 2A). However, corner test showed the attainment of a steady state of neurological functional impairment on day 7 and day 14 following acute IS in groups 2, 3 and 5 but an improvement in neurological function was noted in group 3 as compared to groups 2, 4 and 5. Significant improvement in neurological function became apparent in groups 3 and 4 as compared with group 2, and further improvement was noted in group 5 than in group 2 on day 21 after acute IS (Figure 2B).

### Attenuation of Inflammatory Response through Combined Therapy with CsA and EPO

**Figure 3:**
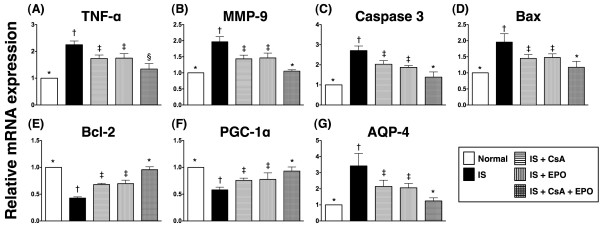
**Profiles of mRNA expression in infarct area**. **A) **Tumor necrosis factor (TNF)-α mRNA expression was remarkably higher in group 2 than in other groups, notably higher in group 3 and 4 than in group 1 and 5, and significantly higher in group 5 than in group 1. † vs. other groups, p < 0.0001 (ANOVA test). **B) **Matrix metalloproteinase (MMP)-9 mRNA expression markedly increased in group 2 than in other groups, notably increased in group 3 and 4 than in group 1 and 5, but no remarkable difference between group 3 and 4 or between group 1 and 5. † vs. other groups, p < 0.0001 (ANOVA test). **C) & D) **Substantially higher mRNA expressions of caspase 3 **(C) **and Bax **(D) **in group 2 than in other groups, and significantly higher in group 3 and 4 than in group 1 and 5, but no notable difference between group 3 and 4 or between group 1 and 5. † vs. other groups, p < 0.0001 (ANOVA test). **E) & F) **Significantly lower mRNA expressions of Bcl-2 and PGC-1α in group 2 than in other groups and markedly lower in group 3 and 4 than in group 1 and 5, but no difference between group 3 and 4 and between group 1 and 5. † vs. other groups, p < 0.0001 (ANOVA test). **G) **Substantially higher mRNA expression of aquaporin-4 (AQP-4) in group 2 than in other groups and remarkably higher in group 3 and 4 than in group 1 and 5, but no significant difference between group 3 and 4 or between group 1 and 5. † vs. other groups, p < 0.001 (ANOVA test). Symbols (*, †, ‡, §) from **A) **to **G) **indicate significance (at 0.05 level) (by Scheffe multiple-comparison post hoc test) (n = 6 for each group).

**Figure 4:**
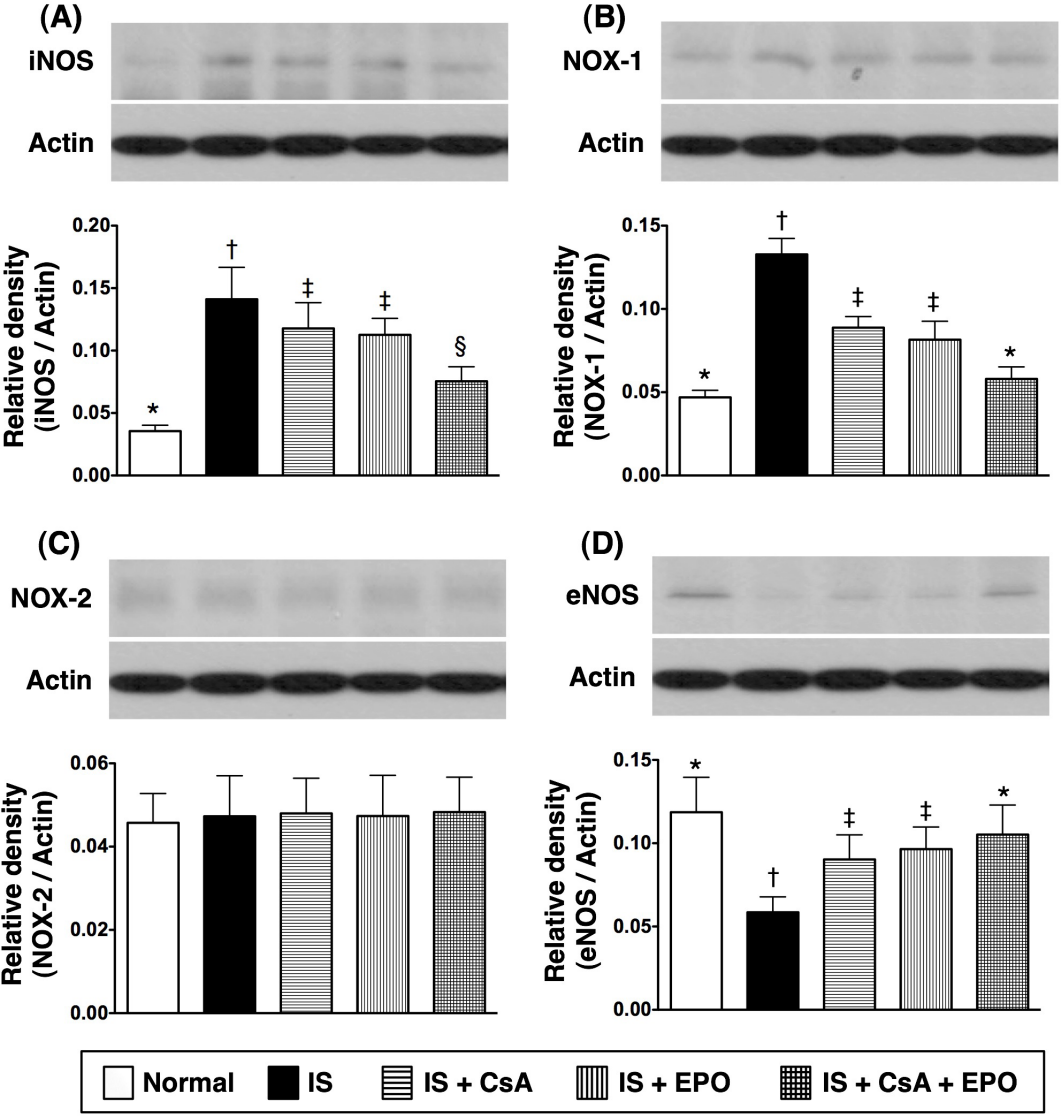
**Protein expression levels of inflammation and oxidative stress-related in infarct area**. **A) and B) **Remarkably elevated protein expressions of inducible nitric oxide synthase (iNOS) **(A) **and NADPH oxidase 1 (NOX-1) **(B) **in group 2 than in other groups, notably higher in group 3 and 4 than in group 1 and 5, significantly increased in group 5 than in group 1, but no difference between group 3 and 4. † vs. other groups, p < 0.001 (ANOVA test). **C) **No significant difference in NOX-2 protein expression among all groups. **D) **Remarkably lower protein expressions of endothelial (e)NOS in group 2 than in other groups, notably lower in group 3 and 4 than in group 1 and 5, but no difference between group 3 and 4. Similar eNOS protein expression noted between group 1 and group 5. † vs. other groups, p < 0.001 (ANOVA test). Symbols (*, †, ‡, §) from **A) **to **D) **indicate significance (at 0.05 level) (by Scheffe multiple-comparison post hoc test) (n = 6 for each group).

On day 21 following acute IS induction, the mRNA expressions of tumor necrosis factor (TNF)-α and matrix metalloproteinase (MMP)-9, two indicators of inflammation, were notably higher in group 2 as compared to other groups (Figure 3, A and 3B). In addition, these two biomarkers were significantly higher in groups 3 and 4 than in groups 1 and 5. Furthermore, TNF-α expression was significantly higher in group 5 as compared with group 1. However, the MMP-9 expression showed no difference between groups 1 and 5. Additionally, the protein expression of inducible nitric oxide synthase (iNOS), an index of inflammation, was remarkably higher in group 2 than in other groups, notably higher in groups 3 and 4 than in groups 1 and 5, and significantly higher in group 5 than in group 1 (Figure 4A). Furthermore, the protein expression of NADPH oxidase 1 (NOX-1), an index of reactive oxygen species (ROS) formation, was significantly higher in group 2 compared to that in other groups and notably higher in groups 3 and 4 than in groups 1 and 5, but it was similar between group 1 and group 5 (Figure 4B). On the other hand, the protein expression of NOX-2 did not differ among the 5 groups (Figure 4C). In contrast, the protein expression of endothelial NOS (eNOS), in index of anti-inflammation, was remarkably lower in group 2 than in other groups, notably lower in groups 3 and 4 than in groups 1 and 5, but no significant difference was noted between group 1 and group 5 (Figure 4D).

### Enhanced Reduction of Apoptosis and Oxidative Stress by Combined Treatment with CsA and EPO

**Figure 5:**
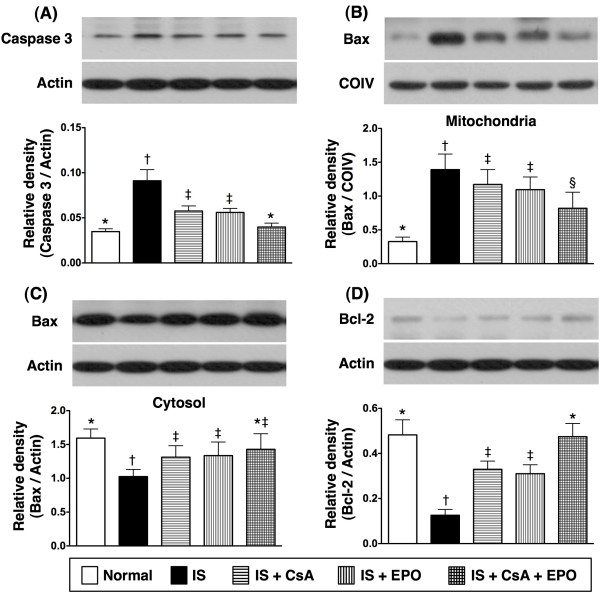
**Protein expression levels of apoptosis-related in infarct area**. **A) **Caspase 3 protein expression was notably higher in group 2 than in other groups, notably higher in group 3 and 4, but no significant difference between group 3 and 4 and between group 1 and 5. † vs. other groups, p < 0.0001 (ANOVA test). **B) **Significantly higher mitochondrial protein expression of Bax in group 2 than in other groups. Significant elevation also noted in group 3 and 4 compared with that in group 1 and 5, and notably higher in group 5 than in group 1, but no remarkable difference between group 3 and 4. † vs. other groups, p < 0.001 (ANOVA test). **C) **Cytosolic protein expression of Bax substantially lower in group 2 than in other groups, but no difference between group 1 and 5 or among group 3, 4, and 5. † vs. other groups, p < 0.001 (ANOVA test). **D) **Bcl-2 protein expression notably lower in group 2 than in other groups, significantly lower in group 3 and 4 than in group 1 and 5, but no significant difference between group 1 and 5 or between group 3 and 4. † vs. other groups, p < 0.001 (ANOVA test). Symbols (*, †, ‡, §) in **A) **to **D) **indicate significance (at 0.05 level) (by Scheffe multiple-comparison post hoc test).

On day 21, the mRNA (Figure 3C) and protein expressions (Figure 5A) of caspase 3, one pro-apoptotic index, were substantially higher in group 2 than in other groups. They were also markedly higher in groups 3 and 4 than in groups 1 and 5, but they did not show significant difference between groups 1 and 5. Additionally, the mRNA (Figure 3D) and mitochondrial protein expressions (Figure 5B) of Bax, another pro-apoptotic index, were substantially higher in group 2 than in other groups, notably higher in groups 3 and 4 than in groups 1 and 5, and the mitochondrial protein expression significantly higher in group 5 than in group 1. However, the Bax mRNA expression only had a statistical trend of notably higher in group 5 than in group 1. On the other hand, the cytosolic protein expression of Bax (Figure 5C) was significantly lower in group 2 than in other groups, notably lower in groups 3 and 4 than in group 1, but it showed no difference between groups 1 and 5 or among groups 3, 4 and 5.

**Figure 6:**
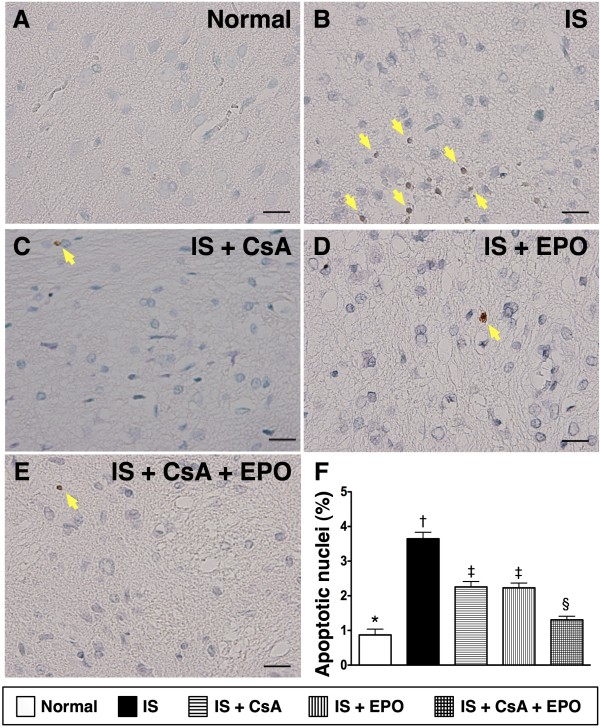
**TUNEL assay for indentifying apoptotic nuclei in brain infarct area**. The number of apoptotic nuclei (yellow arrows) notably higher in group 2 **(B) **than in group 1 **(A)**, group 3 **(C)**, group 4 **(D) **and group 5 **(E)**, significantly higher in group 3 and 4 than in group 1 and 5, and significantly higher in group 5 than in group 1, but no significant difference between group 3 and 4. Scale bars in right lower corner represent 20 μm (400x). † vs. other groups, p < 0.001 (ANOVA test). Symbols (*, †, ‡, §) indicate significance (at 0.05 level) (by Scheffe multiple-comparison post hoc test).

The mRNA (Figure 3E) and protein expressions (Figure 5D) of Bcl-2, an indicator of anti-apoptosis, were notably lower in group 2 than in other groups. The expressions were also significantly lower in groups 3 and 4 than in groups 1 and 5 but without notable difference between groups 1 and 5. Furthermore, TUNEL assay (Figure 6) showed that the number of apoptotic nuclei was substantially increased in group 2 than in other groups, remarkably higher in groups 3 and 4 than in groups 1 and 5, and significantly higher in group 5 than in group 1.

**Figure 7:**
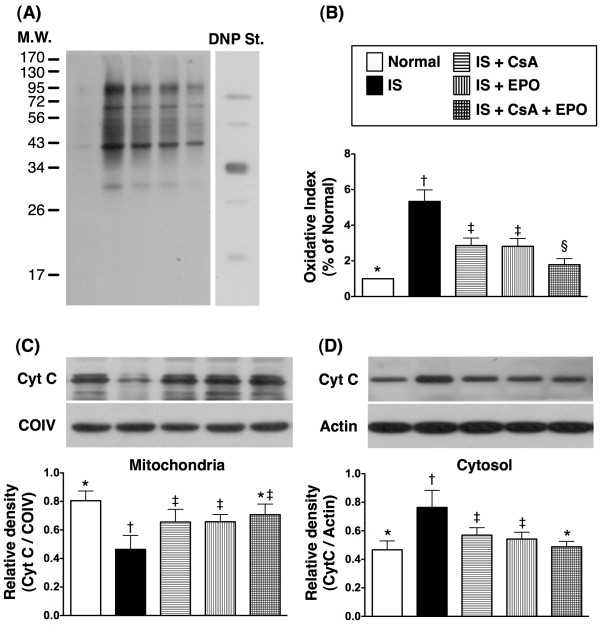
**Oxidative index and protein expression levels of cytochrome (Cyt) C in brain infarct area**. **A) **Oxidative index determination by Western blotting of brain infarct area (BIA) (n = 6), showing notably increased oxidative index, protein carbonyls, in BIA of group 2 compared with other groups, notably higher in group 3 and 4 than in group 1 and 5, and significantly higher in group 5 than in group 1 on day 21 following acute IS. **B) **† vs. other groups, p < 0.0001 (ANOVA test). **C) **Protein expression of mitochondrial cytochrome C in brain infarct area (n = 6) markedly lower in group 2 than in other groups, notably lower in group 3 and 4 than in group 1, but no notable difference among group 3,4, and 5, or between group 1 and 5. † vs. other groups, p < 0.01 (ANOVA test). **D) **Protein expression of cytosolic cytochrome C in BIA (n = 6) markedly higher in group 2 than in other groups, notably higher in group 3 and 4 than in group 1 and 5, but no significant difference between group 3 and 4, or between group 1 and 5. † vs. other groups, p < 0.01 (ANOVA test). Symbols (*, †, ‡, §) from **B) **to **D) **indicate significance (at 0.05 level) (by Scheffe multiple-comparison post hoc test).

On day 21 following acute IS induction, Western blotting (Figure 7, A and 7B) demonstrated a significantly higher oxidative index in mitochondria in group 2 than in other groups. The oxidative index was also significantly higher in groups 3 and 4 than in groups 1 and 5, and notably higher in group 5 compared with that in group 1.

### Better Preservation of Mitochondrial Cytochrome C after Combined Therapy with CS and EPO against Acute IS

The protein expression of cytochrome C in mitochondria (Figure 7C) was significantly reduced in group 2 compared to that in other groups, significantly lower in groups 3 and 4 than in group 1, but it did not differ among groups 3 to 5, or between groups 1 and 5. In contrast, its cytosolic expression (Figure 7D) was significantly enhanced in group 2 compared with other groups, significantly elevated in groups 3 and 4 than in groups 1 and 5, but it did not differ between group 1 group 5. These findings indicate that the expression of cytochrome C, an index of energy supply and storage in mitochondria, was relatively well-preserved in groups 3 to 5 as compared with that in group 2, and was more preserved in group 5 as compared to groups 3 and 4. Additionally, the increase in cytosolic cytochrome C content also suggests significant mitochondrial damage with cytochrome C release into the cytosol in the brain of group 2 animals.

### Further Reduction in Expressions of Glial Fibrillary Acid Protein (GFAP) and Aquaporin-4 (AQP-4) and Preservation of Neural PGC-1α in Infarct Brain after Combined Therapy with CsA and EPO

The mRNA expression of peroxisome proliferator-activated receptor-γ coactivator-1α (PGC-1α) (Figure 3F), which is a transcriptional coactivator for regulating lipid catabolism, oxidative metabolism, mitochondrial metabolism and biogenesis, was notably lower in group 2 than in other groups and significantly lower in groups 3 and 4 than in groups 1 and 5, but it did not differ between groups 3 and 4 or between groups 1 and 5. Conversely, AQP-4 mRNA expression (Figure 3G), an indicator of brain edema, was substantially increased in group 2 compared to that in other groups and notably increased in groups 3 and 4 than in groups 1 and 5, but it was similar between groups 3 and 4 or between groups 1 and 5.

**Figure 8:**
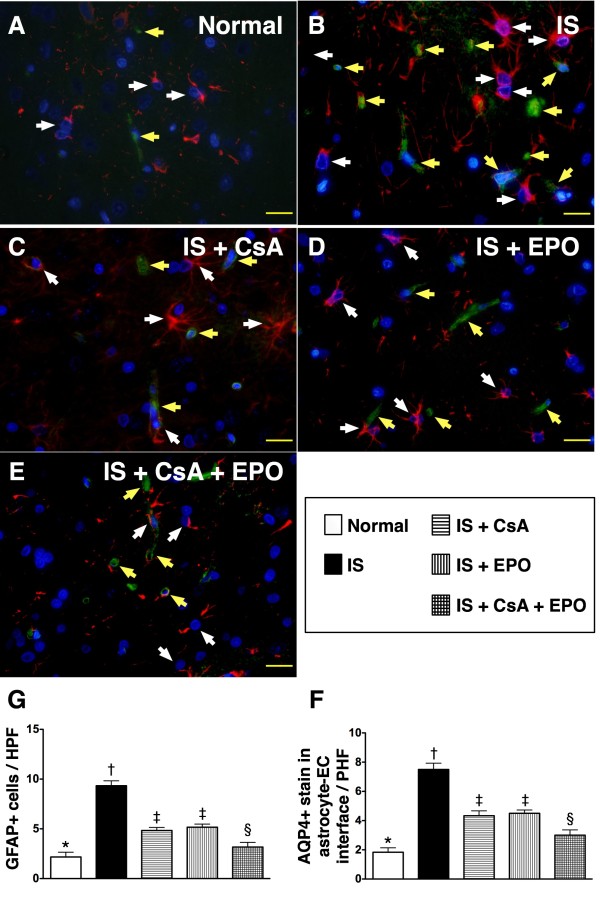
**Distribution of glial fibrillary acid protein (GFAP) and aquaporin-4 (AQP-4) in brain infarct area**. **A) to E) **Immunofluorescent staining (400 x) of glial fibrillary acid protein (GFAP) (white arrows) and aquaporin-4 (AQP-4) (yellow arrows) in brain infarct area (n = 6). Both numbers of GFAG and AQP-4 remarkably higher in group 2 than in other groups, notably higher in group 3 and 4 than in group 1 and 5, and significantly higher in group 5 than in group 1. **F) and G) **† vs. other groups, p < 0.0001 (ANOVA test). Symbols (*, †, ‡, §) in **(F) **and **(G) **indicate significance (at 0.05 level) (by Scheffe multiple-comparison post hoc test). Scale bars in right lower corner represent 20 μm.

**Figure 9:**
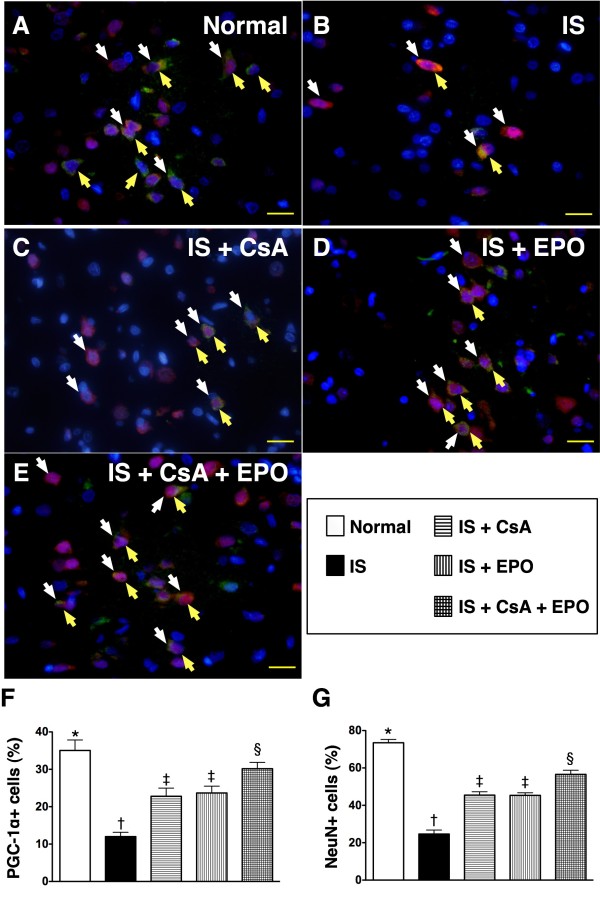
**Distribution of Neural peroxisome proliferator-activated receptor-γ coactivator-1α (PGC-1α) in brain infarct area**. **A) to E) **Immunofluorescent staining (400 x) of PGC-1α (yellow arrows) and neuron (white arrows) in brain infarct area (n = 6). Both numbers of PGC-1α+ cells and neurons remarkably lower in group 2 than in other groups, significantly lower in group 3 and 4 than in group 1 and 5, and significantly reduced in group 5 compared with group 1. **F) and G) **† vs. other groups, p < 0.0001 (ANOVA test). Symbols (*, †, ‡, §) in **(F) **and **(G) **indicate significance (at 0.05 level) (by Scheffe multiple-comparison post hoc test). Scale bars in right lower corner represent 20 μm.

Immunofluorescent staining showed that the expression of GFAP (Figure 8, A-E, white arrows), the principal intermediate filament of mature astrocytes, was remarkably higher (Figure 8G) in group 2 compared to that in other groups, significantly higher in groups 3 and 4 than in groups 1 and 5, and notably higher in group 5 compared to that in group 1. In addition, AQP-4 (Figure 8, A-E, yellow arrows) was substantially increased (Figure 8F) in group 2 than in other groups, notably increased in groups 3 and 4 than in groups 1 and 5, but no significant difference was noted between groups 1 and 5. Conversely, neuronal expression of PGC-1α, an index of mitochondrial integrity (Figure 9, A-E, doubly labeled by yellow and white arrows), was remarkably lower (Figure 9G) in groups 2 than in other groups, notably lower in group 3 and 4 than in groups 1 and 5, and significantly lower in group 5 as compared with that in group 1.

## Discussion

### Combined Therapy with Cyclosporine and EPO Provided Additional Benefits of Limiting Brain Infarct Size and Improving Recovery of Neurological Function

The most important finding in the current study was that TTC staining of the brain tissue on day 21 after acute IS showed that the BIA was remarkably reduced in IS animals treated with either CsA (group 3) or EPO (group 4) than in IS animals without treatment (group 2). These findings imply that CsA or EPO therapy significantly reduce BIA after IS. Moreover, corner test showed a significant improvement in neurological function in groups 3 and 4 than in group 2 on day 21 after acute IS. Interestingly, previous studies [12-15] have demonstrated that EPO therapy significantly reversed ischemia-related left ventricular dysfunction. In concert with this finding, previous investigations by other authors and our recent studies [16,24] have also shown that EPO therapy markedly attenuated BIA and improved neurological function in rat after acute IS. Furthermore, our recent clinical trial [17] has shown that EPO therapy substantially improved 90-day major adverse neurological event. Our findings, therefore, are consistent with those of previous studies [12-17].

Interestingly, as compared with EPO, CsA therapy (group 4) offered similar protection of the brain against infarction/ischemia in the current study. Recent studies [21,22,27,28] have shown that CsA therapy notably reduced infarction size and improved ischemia-related organ function in both animal experiments and clinical observational studies. Thus, our findings strengthen those of the studies [21,22,27,28]. Of importance is that, as compared with those group 3 and group 4, combined therapy with CsA and EPO (group 5) further attenuated BIA. These findings may explain the enhanced improvement in neurological function in group 5 animals as compared with those in group 3 and group 4. In this way, the results of the present investigation extend the findings of previous studies [12-17,21,22,27,28].

Combined therapy with EPO and tissue plasminogen activator (tPA) for patients after acute IS has been recently reported by Ehrenreich et al. [29]. Failure in demonstrating additional benefits of combining EPO with tPA in improving clinical outcome of patients with acute IS as compared with placebo-controls in that clinical trial [29] may be due to tPA-associated bleeding complication that outweighed the benefit of EPO treatment [17].

### Combined Therapy of CsA and EPO Further Limited Inflammatory Reaction, Generation of Reactive Oxygen Species, and Oxidative Stress after Acute IS

Abundant studies have shown that innate immune mechanisms are rapidly activated following acute arterial obstructive syndrome (i.e. tissue injury and necrosis) which, in turn, initiate the complement cascade, inflammatory reaction, and ROS generation [16,23,30,31]. Additionally, inflammatory components of the ischemic cascades further perpetuate cellular apoptosis and necrosis in ischemic region [15,16,22,23,30-33]. One important finding of the present study is that the inflammatory responses were markedly increased in group 2 animals than in those in groups 3 to 5 on day 21 after acute IS. Moreover, both ROS generation (NOX-1) and oxidative stress were remarkably enhanced in group 2 animals than in other groups on day 21 after acute IS. Another intriguing finding of the current study is that the expressions of anti-inflammatory protein, eNOS, was substantially reduced in group 2 than in other groups. Additional important finding also includes the remarkably increased number of GFAP-positive cells, an indicator of inflammatory cells in ischemic brain, in group 2 and notable reduction in groups 3 to 5 after treatment. Therefore, our findings, in addition to corroborating those of previous reports [15,16,22,23,30-33], could at least partially explain the poorer prognostic outcome in group 2 animals compared with those in groups 3 to 5. Besides, the results of our study may support the proposal that both CsA and EPO therapy are equally effective in protecting the brain against ischemic damage after acute IS through suppressing inflammation, generation of ROS, and oxidative stress. Of importance is that combined therapy with CsA and EPO was found to be superior to either one alone in inhibiting the production of inflammatory biomarkers, ROS, and oxidative stress.

### Possible Mechanisms of CsA and EPO Underlying Improved Outcome after Acute Ischemic Stroke

The key role of EPO therapy in improving outcome after acute IS has been mainly attributed to attenuation of inflammation, oxidative stress, cellular apoptosis, and enhancement of angiogenesis and neurogenesis [16,17,24,34]. On the other hand, inhibition of inflammation, oxidative stress, cellular apoptosis, and mPTP opening have been proposed to be the underlying mechanisms involved in CsA-mediated protection against ischemia-reperfusion organ dysfunction [18-20,27,28]. In the current study, not only were the inflammatory and oxidative cascades found to be substantially diminished, but the apoptotic markers were also substantially attenuated after CsA and EPO therapy. Accordingly, the anti-apoptotic index (Bcl-2) was notably enhanced following combined therapy. In addition, reduction in the number of AQP4+ cells and preservation of the number of PGC-1α+ neurons in BIA were observed after CsA and EPO treatment. Moreover, mitochondrial cytochrome C was better preserved in treated than in untreated animals after acute IS. Therefore, our findings not only extend those of previous studies [16-20,24,27,28,34], but they also provide novel information on the superiority of combined therapy with CsA and EPO compared with either agent alone in the treatment of acute IS in an experimental setting. In consideration of the fact that both EPO and CsA are frequently and separately used in our daily clinical practice for variety of disease entities, this pre-clinical information may warrant the need for a prospective clinical trial in evaluating the benefit of combined therapy with CsA and EPO which have been widely used in different clinical settings after acute IS.

### Study Limitation

This study has limitations. First, since the current study period was only 21 days, the long-term effect of combined therapy with CsA and EPO on sensorimotor function in this experimental setting is unknown. Second, this study did not investigate the safety of CsA dosage so that the side-effects of CsA therapy remain unclear. A balance between the benefits and risks of CsA use, therefore, is still a major concern regarding the clinical use of CsA in the setting of acute IS.

## Conclusion

**Figure 10:**
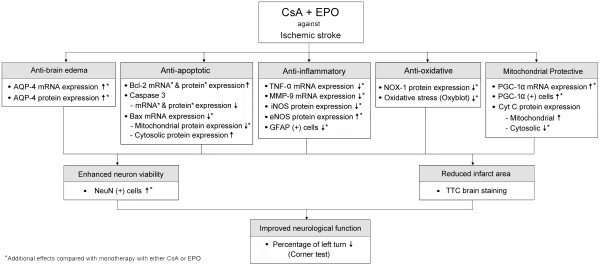
**The proposed mechanisms underlying the protective actions of cyclosporine and erythropoietin in rats after ischemic stroke**.

The results of the present study suggest that combined therapy with CsA and EPO is superior to either agent alone in reducing BIA and improving neurological function after acute IS. The proposed mechanisms underlying the potential impacts of combined CsA and EPO in rats after IS have been summarized in Figure 10.

## Competing interests

The authors declare that they have no competing interests.

## Authors' contributions

All authors have read and approved the final manuscript.

CMY, CKS, YCL, SL, and HKY designed the experiment, performed animal experiments, and drafted the manuscript. LTC, YHK, CHY, YLC, THT and PLS were responsible for the laboratory assay and troubleshooting. SC, CKS, SL, and HKY participated in refinement of experiment protocol and coordination and helped in drafting the manuscript.
